# Syringe Free Vaccination with CAF01 Adjuvated Ag85B-ESAT-6 in Bioneedles Provides Strong and Prolonged Protection Against Tuberculosis

**DOI:** 10.1371/journal.pone.0015043

**Published:** 2010-11-29

**Authors:** Dennis Christensen, Thomas Lindenstrøm, Gijsbert van de Wijdeven, Peter Andersen, Else Marie Agger

**Affiliations:** 1 Statens Serum Institut, Department of Infectious Disease Immunology, Vaccine Delivery and Formulation Research, Copenhagen, Denmark; 2 Bioneedle Technologies Group BV, Eindhoven, The Netherlands; Fundació Institut Germans Trias i Pujol, Universitat Autònoma de Barcelona, CibeRES, Corporate Research Program on Tuberculosis (CRP-TB), Spain

## Abstract

Bioneedles are small hollow sugar based needles administered with a simple compressed air device. In the present study we investigate how incorporation of a subunit vaccine based on TB vaccine hybrid Ag85B-ESAT-6 adjuvated with CAF01 into Bioneedles affects its immunogenicity as well as its ability to protect against TB in a mouse model. The CMI response measured by IFN-γ and antigen specific CD4+ T-cells was, two weeks after the last vaccination, significantly lower in the group immunized with Bioneedle-incorporated vaccine compared to the conventional vaccine, using syringe and needle. However, at four, nine and 52 weeks after vaccination we observed similar high IFN-γ levels in the Bioneedle group and the group vaccinated using syringe and needle and comparable levels of antigen specific T-cells. Furthermore, the protective efficacy for the two vaccination methods was comparable and similar to BCG vaccination both six and 52 weeks after vaccination. These results therefore advocate the further development of the Bioneedle devises and applicators for the delivery of human vaccines.

## Introduction

Parenteral injection using needles and syringes is the most common route of administration of vaccines today. However this method possesses safety risks for patients and health care providers. A recent publication showed e.g. that 72.6% of the healthcare staff in Kabul hospitals reported sharps injuries over a period of 12 months [1]. Consequently the number of infections among health care professionals attributable to injuries from needles and syringes are worldwide estimated to 66,000 hepatitis B (HBV) incidences, 16,000 hepatitis C virus (HCV) incidences and 1,000 human immunodeficiency virus (HIV) incidences per year [2]. Furthermore, improper vaccination practice, such as reuse of needles and syringes are commonly found in the developing world, resulting in an increased risk for pathogen transmission during vaccination. In a comprehensive study conducted in Africa in 2000, WHO found that reuse of injection equipment in the absence of sterilization, was only assumed to have zero risk in four out of 14 regions. In the remaining 10 regions, WHO estimated that 1.2% to 75% of the annual 1.7–11.3 injections per person were administered with reused equipment causing an estimated 21 million HBV infections, two million HCV infections and 260 000 HIV infections [3].

With the high prevalence of diseases like HBV, HCV and HIV in tuberculosis (TB) endemic regions it is of crucial importance to consider safe delivery strategies when developing novel vaccines against TB. Even though much effort has been devoted to developing alternative administration techniques for vaccines, there is still a need for a safe, effective needle free vaccine delivery system, which at the same time is easy to distribute and handle even in rural districts of the developing world, with lack of reliable roads, refrigeration and educated personal. One potential approach is transcutaneous immunization, with the vaccine being delivered to the immune cells of the skin, usually by using a patch for hydration and/or disruption of the stratum corneum allowing penetration of the vaccine [4], [5], [6]. However, there is limited experience about their applicability in the developing world, where it can be more difficult to monitor compliance issues i.e. that the patches are kept on for the appropriate amount of time and that they are kept dry and clean. In order to obtain high compliance it is in general a much more safe approach to apply a vaccine in a manner that prevents the vaccinated in discontinuing the immunization. This on the other hand relies on educated personal and simple to handle devises which are easy to repair or replace. Jet injectors delivering the vaccine to the intradermal, subcutaneous or intramuscular cavities through high pressure nozzles have been implemented with variable success in mass immunization campaigns. A Hepatitis B outbreak in 1985 was linked to the use of multi-use-nozzle jet injectors that were contaminated by body fluids during vaccination, thereby causing a disease spread [7]. This incidence halted the use of multi-use-nozzle jet injectors and led to the development of new devises with disposable caps [8] or single-use-nozzle devises [9]. A major draw-back of these devises is that there is no international standard on vaccine cartridges hindering the application of different devises for the same vaccine, which for practical reasons usually only will be filled in one type of containers. Another draw-back is that these devises are often highly sophisticated electrical equipment, which makes them inappropriate for use in the developing world.

**Figure 1:**
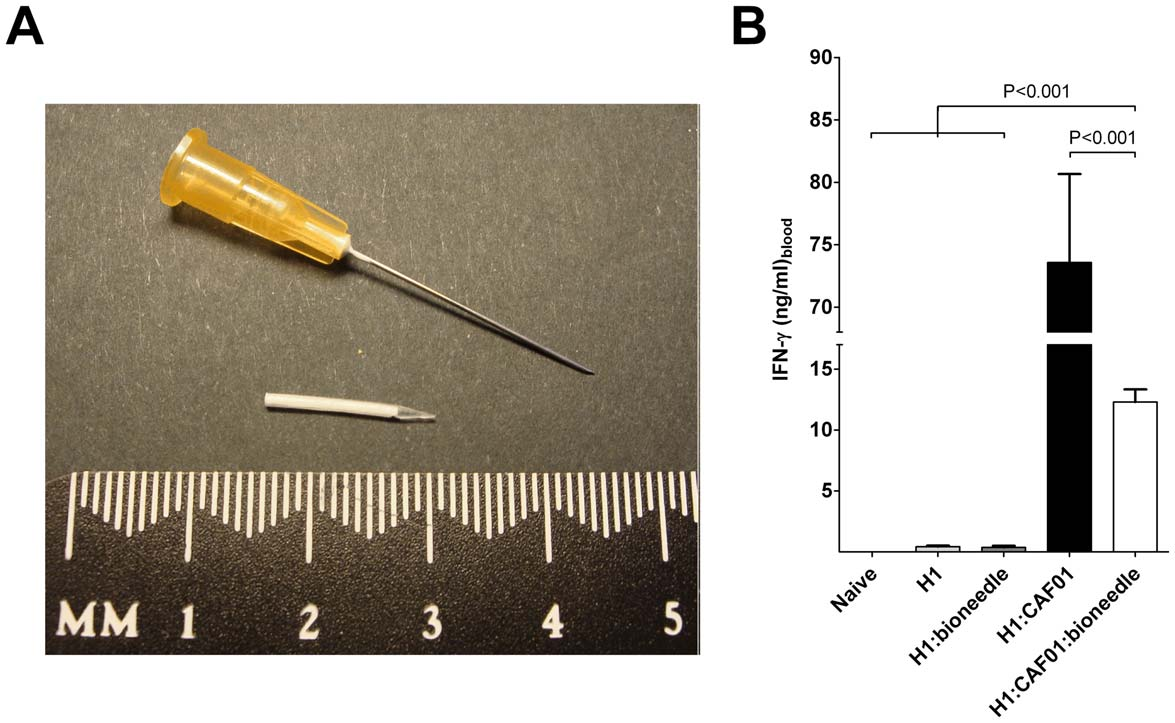
Bioneedles are small hollow sugar based needles. A Bioneedle is pictured in A) next to a standard 0.5×25 mm Terumo needle. B) IFN-γ responses in mice (pooled PBMCs) two weeks after the last of three immunizations with 2 µg of H1 antigen alone (light grey) or in Bioneedles (dark grey), combined with CAF01 (black) or combined with CAF01 and incorporated in Bioneedles (white).

An interesting new approach is to inject mini implants containing the vaccine into the body using a low-tech compressed air devise. One such technology is Bioneedles, which are small hollow sugar based needles (Figure 1A) administered into the muscle with a simple compressed air devise from an initial distance of 3–10 mm from the skin surface, thereby avoiding cross contamination of patients [10]. The wound at site of injection is no bigger than that observed with an ordinary needle and since the duration of the vaccination is only approximately 0.7 milliseconds, faster than the time it takes to recognize pain, then the vaccine has already been administered by the time the vaccinated realizes it, practically abolishing the feeling of pain (van de Wijdeven; publication in preparation). Bioneedles are prepared of polymers which upon administration will be degraded by enzymes resulting in a release of the vaccine. They do, however, not contain any adjuvant effect making it necessary to combine the vaccine antigen with an adjuvant to induce the appropriate immune response. Bioneedles has previously been tested together with a AlPO_4_ adjuvated tetanus toxoid vaccine [11] in a mouse model. This study showed that those injected with the vaccine in a bioneedle obtained a functional antibody response comparable to the group receiving the conventional liquid vaccine.

In the present study we investigate how incorporation of a subunit vaccine based on adjuvated TB vaccine hybrid Ag85B-ESAT-6 (H1) into Bioneedles affects its immunogenicity as well as its ability to protect against TB in a mouse model. Numerous studies have demonstrated a positive correlation between the induction of a Th1 response and resistance to a subsequent challenge with TB [12]. This excludes the use of any of the adjuvants presently approved for human use all being Th2 inducers, whereas previous studies have shown that CAF01 (also designated DDA/TDB) is capable of inducing protective Th1 immune responses against TB when combined with H1 [13], [14]. H1 is currently in phase two clinical trials in combination with IC31 adjuvant, as well as phase one clinical trials with the CAF01 adjuvant.

With the pros and cons related to the use of needles and syringes in mind, we have to consider new vaccination concepts which can fulfil the requirements to safety, stability, ease-of-application and reliability. These aspects are of particular importance when developing a new vaccine against TB and the Bioneedle technology fulfils these criteria. The combination of a novel TB vaccine with the Bioneedle delivery system, therefore offers a unique modern vaccination concept. In the present study we therefore investigated how incorporation of H1 and CAF01 into Bioneedles affects the protective efficacy in mice as compared to a similar vaccine injected with syringe and needle. We found, that even though the immune response was altered, we still obtained protective efficacy comparable to both the conventional vaccine using syringe and needle and to the existing BCG vaccine.

## Results and Discussion

### Incorporation of the vaccine into Bioneedles resulted in a delayed immune response as compared to the regular vaccine

In this study, a panel of vaccines containing tuberculosis vaccine antigen H1 alone or in combination with Bioneedle, CAF01 or CAF01:Bioneedle was used for vaccination and the CMI response evaluated. Three injections were given s.c. to C57BL/6 mice with a two-week interval and blood samples were collected by periorbital puncture at week 2, 4, 9 and 52 after the last vaccination. The levels of IFN-γ secretion were assessed by ELISA analysis upon *in vitro* restimulation with H1. Vaccination with the Ag alone or Ag:Bioneedle did not increase the immune response against the antigen (Figure 1). These groups were therefore not included in the long term memory and -protection studies.

At the early time point, two weeks after the last immunization, incorporation of the adjuvated vaccine into the Bioneedle (Ag:CAF01:Bioneedle) reduced the vaccine immunogenicity as compared to the conventional vaccine (Ag:CAF01). This is observed by a significant reduction of IFN-γ cytokine production of Ag:CAF01:Bioneedle as compared to Ag:CAF01 (Figure 1B).

**Figure 2:**
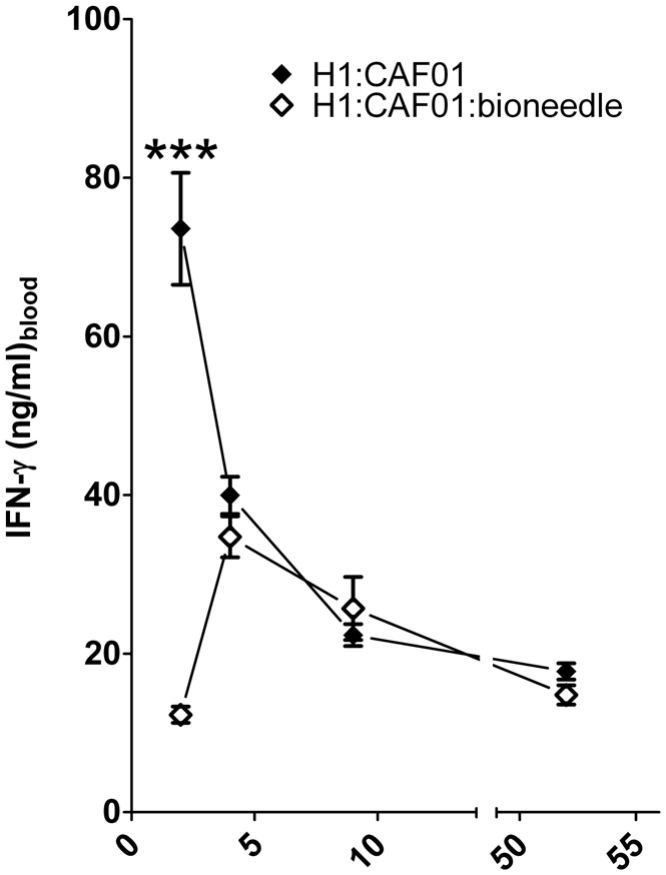
IFN-γ responses in mice. The IFN-γ responses were measured upon restimulation of pooled PBMCs 2, 4, 9 and 52 weeks after the last of three immunizations with 2 µg of H1 antigen combined with CAF01 (black) or combined with CAF01 and incorporated in Bioneedles (white). Values marked with an asterisk are significantly different (***, p<0.001).

This significant reduction was not observed after 4, 9 and 52 weeks which could be explained by a delayed release of the vaccine from the Bioneedles giving an equally delayed immune response as compared to regular vaccination (Figure 2). The endpoint data after restimulation of spleenic cells obtained 52 weeks after vaccination was comparable to those obtained in PBMCs (data not shown).

#### Bioneedle embedded vaccine resulted in altered frequency of highly differentiated T-cells as compared to the regular vaccine

**Figure 3:**
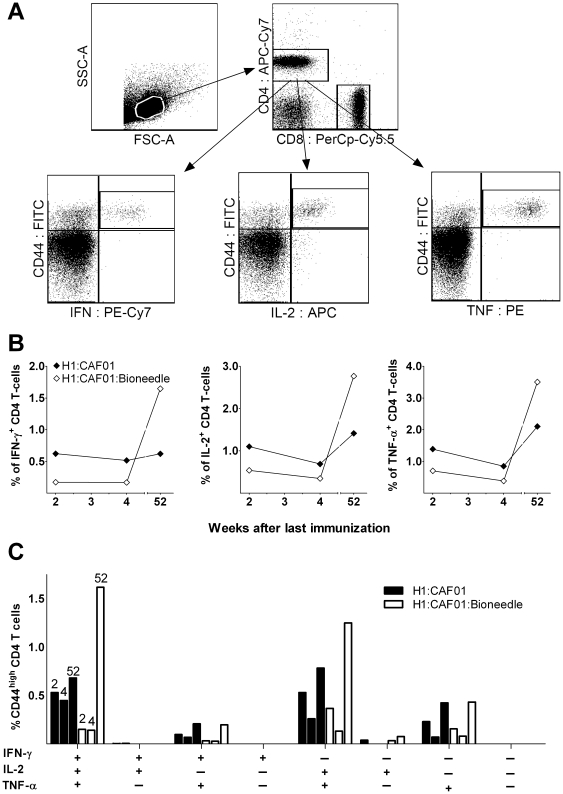
An icFACS analysis of cytokine expression in CD4 T cells in mice. The cytokine expression pattern were measured upon restimulation of pooled PBMCs 2, 4 and 52 weeks after the last of three immunizations with 2 µg of H1 antigen combined with CAF01 (black) or combined with CAF01 and incorporated in Bioneedles (white). A) Gating tree for phenotypic and functional characterization of memory CD4 T cells in H1:CAF01-immunized mice by multiparameter flow cytometry. Lymphocytes were gated based on their forward scatter (FSC) vs side scatter SSC profile, and the CD4+CD44high. B) Intracellular IFN-γ, TNF-α, and IL-2 levels expressed as geometric MFI values were compared within the CD4+CD44high population. There were no significant difference between the MFI values obtained from the two vaccination groups. C) The cytokine-producing cells were divided into seven distinct subpopulations based on their production of these cytokines in any combination. The bar chart shows the frequency of each cytokine subset being CD44high out of the total CD4 T cell population. The bars within each subpopulation represent the two vaccination methods at week two, four and 52 after vaccination (exemplified in the triple positive subpopulation).

The ability of T_H_1 cells to produce IFN-γ has been described as essential to drive an effector T-cell response, whereas TNF-α and especially IL-2 are necessary for the additional induction of a memory T-cell response [15], [16]. The IFN-γ results described previously suggest that the magnitude of the vaccine induced T-cell response observed in blood two weeks after immunization is lower when administered in Bioneedles as compared to the regular vaccine using syringe and needle. We therefore characterized the phenotype and cytokine production of the T-cells using intracellular flow cytometry staining after immunization with H1 combined with either CAF01 or CAF01:Bioneedle. H1 specific CD44^high^ CD4 T cells were assessed for their cytokine production to establish the proportion of CD4 cells expressing IFN-γ, TNF-α, and IL-2. The CD4^+^CD44^high^ T-cell population was separated into seven distinct subpopulations based on their production of IFN-γ, IL-2, or TNF-α in any combination, and the representation of each of these subpopulations within the pool of T cells was established (Figure 3).

The CD4 T cells induced by CAF01 two weeks after the last vaccination were primarily IFN-γ+TNF-α+IL-2+ multifunctional T cells and TNF-α+IL-2+ central memory T cells (∼37% for each subpopulation). The overall induction of IFN-γ-, TNF-α and/or IL-2 producing T-cells was reduced when using Bioneedles for vaccination at this early timepoint after vaccination (Figure 3B). This was especially the case for the highly differentiated IFN-γ+TNF-α+IL-2+ T-cells which only accounted for ∼20% whereas the less differentiated TNF-α+IL-2+ cell population made up ∼50% of the total population. Two weeks later (week four post vaccination) the overall T-cell frequency had waned a little for both vaccine groups but the T-cell differentiation pattern was similar to the previous results. An increase in the frequency of IFN-γ-, TNF-α and/or IL-2 producing T-cell was observed from week 4 to week 52 post vaccination with both the Bioneedle embedded and the regular vaccine. The increase was however most pronounced in the CAF01:Bioneedle vaccinated animals, where at least an 8-fold increase in the frequency of cytokine producing T-cells was observed (Figure 3B). The multifunctional cells that express TNF-α+IL-2+ double positive and IFN-γ+TNF-α+IL-2+ triple-positive cells dominated the responding T cell population in both vaccination groups (Figure 3C). At all timepoints, the responses in naive control mice were undetectable (data not shown) regardless of the cytokine measured. CD8 responses were likewise evaluated but no CD8-specific responses were observed in any of the groups. The endpoint data after restimulation of spleenic cells obtained 52 weeks after vaccination was comparable to those obtained in PBMCs (data not shown).

CAF01 has in previous studies been shown to form a vaccine depot at site of injection [17]. This depot effect could very well be even more pronounced with the condensation of the vaccine and its incorporation into the Bioneedles, which could well explain the increase in cytokine producing T-cells at the late time point after vaccination with the Bioneedle embedded vaccine (Figure 3B).

#### Bioneedle embedded vaccine induced protection against TB comparable to the regular vaccine and BCG

**Figure 4:**
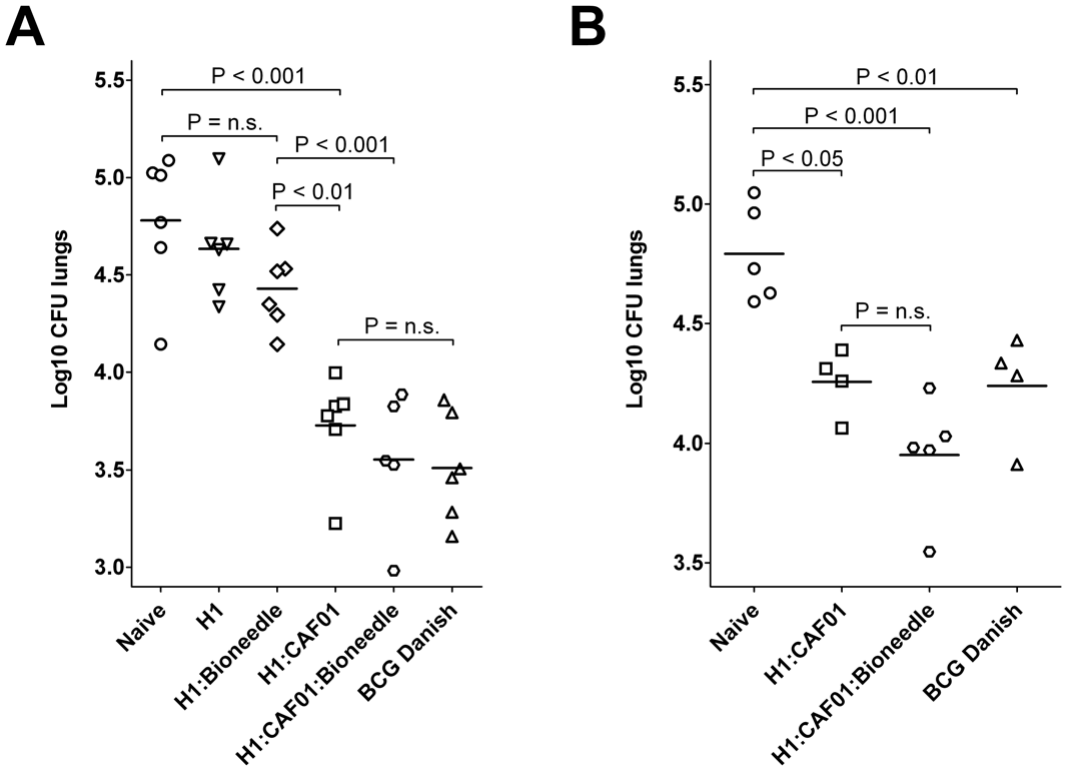
TB bacterial burden in mice upon vaccination. Mice were challenged by the aerosol route with virulent M. tuberculosis A) 6 weeks and B) 52 weeks after the last immunization. Six weeks post challenge, mice were sacrificed and the bacterial burden (CFU) measured in the lungs (expressed as log10 CFU). As a positive control group, a group of mice received a BCG vaccination ten weeks before challenge.

The ability of the CAF01 adjuvated H1 vaccine delivered in Bioneedles to elicit protection against *M. tuberculosis* was then evaluated and compared to protection levels obtained with unvaccinated and BCG vaccinated mice. The mice were challenged through the aerosol route with *M. tuberculosis* Erdman. H1 in CAF01 and CAF01:Bioneedles provided significant levels of protection in the lungs (p<0.001) comparable to that offered by the standard BCG vaccination with approximately one log reduction in bacterial numbers (Figure 4A). As expected, vaccination with H1 alone or delivered in Bioneedles, resulting in low levels of cell-mediated immune responses, also failed to provide protection against TB. We next assessed whether the memory CMI obtained after vaccination with H1 combined with either CAF01 or CAF01:Bioneedle (Figure 2 and 3) was translated into protective efficacy by challenging mice at 52 weeks post vaccination. Even one year after vaccination the H1 in CAF01 and CAF01:Bioneedles were still able to induce approximately half a log reduction in bacterial numbers, which was comparable to that seen in age matched mice receiving the live BCG vaccine six weeks prior to challenge (Figure 4B). Furthermore, a slightly albeit not significant, increase in protection was observed when administering H1 in CAF01:Bioneedles as compared to CAF01, correlating with the increased amount of memory T-cells obtained at this late timepoint.

These results therefore suggest that the administration of H1:CAF01 TB vaccine in Bioneedles is at least as good if not better at inducing protective long term memory against TB as the same vaccine administered with needle and syringe as well as the presently approved BCG vaccine. Combined with the advantages regarding logistics (low sample weight and volume), no need for preservatives, ease of use, low pain and – maybe most importantly – that the method permits the administration without contacting the skin, thus avoiding cross contamination between handlings [10] advocate the further development of the Bioneedle devises and applicators for the delivery of human vaccines.

## Materials and Methods

All the necessary permits for this study were provided by the Danish Ministry of Justice.

### Materials

DDA (purity >99%) and TDB (purum >99%) were obtained from Avanti Polar Lipids (Alabaster, AL). Methanol (extra pure), chloroform (extra pure) and 1 M hydrochloric acid, used to adjust pH in the Tris-buffer, were purchased from Merck (Darmstadt, Germany). Tris base (99%) and cholesterol (purity ≥ 99%) were obtained from Sigma-Aldrich Inc. (St. Louis, MO). Water for injection (WFI) from Statens Serum Institut (Copenhagen, Denmark) was used to prepare the buffer.

### Preparation of CAF01 liposomes

CAF01 was formulated by the thin film method as previously described [18]. Weighed amounts of DDA and TDB were dissolved in chloroform/methanol (9∶1, by volume) and the organic solvent was removed using a gentle stream of N_2_ forming a thin lipid film on the bottom of the test tube. The lipid film was dried over night to remove trace amounts of the organic solvent. The lipid film was hydrated in 10 mM Tris-buffer at pH 7.4, to a final concentration of 5.0 mg/ml DDA and 1.0 mg/ml TDB, by heating for 20 min at 60°C with in-between whirl mixing.

### Preparation of vaccine in Bioneedles

Bioneedles were produced according to Bioneedle Technologies Group's proprietary procedures using injection molding of thermoplastic starch. In order to incorporate an entire mouse dose of CAF01 (0.25 mg DDA +0.05 mg TDB) into the inside cavity of the Bioneedles with a volume of about 4.0–4.6 µl it was necessary to reduce the dose volume with a factor 12.5. This was done by vacuum concentration for 10 minutes using a Heto Hetovac VR-1 and Heto CT110 vacuum concentrator followed by heating to 60°C for 5 minutes on a water bath and whirl mixing. This procedure was repeated until the dose volume was 4.0 µl. 2 µg of the vaccine antigen H1, produced as previously described [19], was added before up-concentration. The concentrated vaccines were transferred to a 1 ml BD syringe for filling the Bioneedles. Bioneedles containing 2 µg/dose of H1 was prepared for reference in the immunization experiments. After filling, the Bioneedles were frozen to −55°C on the temperature controlled shelf in a Christ ALPHA 2–4 LD freeze-dryer (Martin Christ, Osterode am Harz, Germany) followed by freeze drying. The primary drying was carried out through 20 hours at −25°C and 4 mBar vacuum. The shelf temperature was then gradually raised to +20°C over a period of 20 hours whilst in the same time decreasing pressure until 0.04 mbar. A final drying step was done at +20°C and 0.04 mBar for 6 hours. After filling and freeze-drying, the Bioneedles were individually stored in vials, closing these under ambient air and relative humidity, using rubber stoppers and aluminium caps.

### Vaccination of mice

The study is build on two experiments: 1) a long term immunogenicity experiment in which the mice were challenged with TB 52 weeks after the last immunization and a short term TB challenge study. Female C57BL/6 mice, 6–12 weeks old, obtained from Harlan Scandinavia (Allerod, Denmark) were divided into groups of eight for the investigation of immunogenicity and groups of six for the short term TB challenge study. The mice receiving the regular injections were immunized three times with a 2-week interval, subcutaneously (s.c.) at the base of the tail with 0.2 ml vaccine containing either 2 µg H1 alone or in combination with CAF01 (0.25 mg DDA +0.05 mg TDB). The mice receiving the Bioneedle vaccinations were immunized simultaneously with those receiving the regular injections. The Bioneedles were injected in the scruff of the neck using a disposable one-way implanter (Loligo Systems, Tjele, Denmark). A group of BCG-vaccinated mice was included in each TB challenge experiment as a control for vaccine efficacy. These mice received 5×10^6^ CFU of BCG Danish 1331 s.c. in 0.2 ml of 0.9% saline at the same time that the remaining test groups received their first immunization with experimental subunit vaccines. No booster injections were given to the BCG group. All experiments were conducted in accordance with the regulations of the Danish Ministry of Justice and animal protection committees (Permit no. 2004/561-868) and in compliance with European Community Directive 86/609.

### Immunogenicity assays

#### Enzyme-linked immunosorbent assay (ELISA)

Blood was obtained by periorbital puncture and the peripheral blood mononuclear cells (PBMCs) were purified as previously described [20]. All cell cultures were performed in microtitre plates (Nunc, Roskilde, Denmark) containing 2×10^5^ cells in a volume of 200 µl RPMI-1640 supplemented with 5×10^5^ M 2-mercaptoethanol, 1 mM glutamine, 1% pyruvate, 1% penicillin–streptomycin, 1% HEPES, and 10% fetal calf serum (all from Gibco Invitrogen, Carlsbad, Denmark). PBMCs isolated two, four and 52 weeks post vaccination were stimulated with H1-antigen at a concentration of 0.5 µg/ml. Wells containing medium only or 5 µg/ml concanavalin A were used as negative and positive controls, respectively. Culture supernatants were harvested after 72 hr incubation and the amounts of IFN-γ were determined by ELISA as previously described [21].

#### Intracellular flow cytometric analysis (icFACS)

PBMCs isolated two, four and 52 weeks post vaccination were stimulated with 5 µg/ml H1 for 1 h in the presence of 1 µg/ml anti-CD28 (clone 37.51) and anti-CD49d (clone 9C10 (MFR4.B); both from BD Pharmingen) and subsequently incubated for 5–6 h at 37°C following the addition of 10 µg/well brefeldin A (Sigma-Aldrich) and 0.7 µl/well monensin/GolgiStop (BD Pharmingen). Following overnight storage at 4°C, cells were washed in FACS buffer (PBS containing 0.1% sodium azide and 1% FCS), and subsequently stained for 30 min at 4°C for surface markers with mAbs as indicated using 1/200 dilutions of anti-CD4-allophycocyanin-Cy7 (clone GK1.5), anti-CD8-PerCP-Cy5.5 (clone 53-6.7), and anti-CD44-FITC (clone IM7) (all BD Pharmingen). Cells were then washed in FACS buffer, permeabilized using the Cytofix/Cytoperm kit (BD Pharmingen) according to the manufacturer's instructions, and stained intracellularly for 30 min at 4°C in dilutions of 1/100 using anti-IFN-γ-PE-Cy7 (clone XMG1.2; eBioscience), anti-TNF-α-PE (clone MP6-XT22; BD Pharmingen), or anti-IL-2-allophycocyanin (clone JES6-5H4; BD Pharmingen) mAbs. Cells were subsequently washed, resuspended in FACS buffer, and then analyzed using a six-colour BD FACSCanto flow cytometer (BD Biosciences). Finally, responses were analyzed using FlowJo software version 8.7 (Mac) followed by Pestle and Spice (Tree Star).

### TB challenge of mice

*M. tuberculosis* Erdman for infection was grown in suspension in modified Sauton medium enriched with 0.5% sodium pyruvate, 0.5% glucose and 0.02% Tween 80 [20]. Challenge infections with *M. tuberculosis* were administered six or 52 weeks after the last immunization, by the aerosol route in a Glas-Col inhalation exposure system (Glas-Col, Terre Haute, IN) with an inoculum of ∼25 CFU per mouse. The mice were sacrificed after six weeks of challenge infection, and bacterial numbers in the lungs were determined by macerating the organs and plating serial threefold titrations on Middlebrook 7H11 agar plates. Organs from BCG-vaccinated mice were grown on 7H11 plates supplemented with 2 µg of 2-thiophenecarboxylic acid hydrazide per ml to selectively inhibit the growth of BCG. After 2 weeks of incubation at 37°C, the numbers of bacteria were counted and reported in CFU. All results are based on individual analyses of five to six mice per group.

### Statistical analysis

For comparative analysis of immunogenicity, data were tested by one-way analyzes of variance (ANOVA). When significant differences were indicated, differences between means were determined by Bonferroni's multiple comparison test. All statistical analyses were performed in GraphPad Prism version 5 (GraphPad Software Inc., La Jolla, CA).
